# Towards a Treatment for Intolerance of Uncertainty in Young People with Autism Spectrum Disorder: Development of the Coping with Uncertainty in Everyday Situations (CUES©) Programme

**DOI:** 10.1007/s10803-016-2924-0

**Published:** 2016-10-28

**Authors:** Jacqui Rodgers, Anna Hodgson, Kerry Shields, Catharine Wright, Emma Honey, Mark Freeston

**Affiliations:** 1 Clinical Psychology, Institute of Neuroscience, Faculty of Medical Sciences, Newcastle University, Ridley Building, Newcastle upon Tyne, NE1 7RU UK; 2 School of Psychology, Faculty of Medical Sciences, Newcastle University, Newcastle upon Tyne, UK; 3 Northumberland, Tyne and Wear NHS Foundation Trust, Newcastle upon Tyne, UK; 4 Northumbria Healthcare NHS Trust, North Shields, UK

**Keywords:** Anxiety, Intolerance of uncertainty, Parent group, Intervention

## Abstract

Intolerance of uncertainty (IU) is indicated as an important transdiagnostic process variable in a range of anxiety disorders. Anxiety is very common in children with autism spectrum disorders (ASD). This study aimed to develop a parent group based manualised treatment programme for young people with ASD, which focused on IU. An eight session programme was developed and then delivered to 11 parents across three treatment groups, two recruited via a research data base and one via clinical services. Data regarding retention, acceptability and feasibility indicate that the parents valued the programme. Effect size analyses of outcome measures for potential use in larger trial indicate that the programme has promise as a treatment option of your people with ASD and IU.

## Background

A significant number of individuals with autism spectrum disorders (ASD) experience co-occurring anxiety. It is estimated that around 50 % of children with ASD will experience significant anxiety (Simonoff et al. 2008). Anxiety is a frequent reason for families to seek help from NHS services. The presence of anxiety symptoms in childhood is a significant predictor of the development of an anxiety disorder in adulthood indicating the long-term psychological, social and economic significance of addressing childhood anxiety in ASD. Individuals with ASD frequently experience multiple anxiety disorders concurrently, therefore treatments targeting underlying mechanisms may be most efficacious.

*Intolerance of uncertainty* (IU) is a “dispositional risk factor for the development and maintenance of clinically significant anxiety” in neurotypical populations (Carleton 2012). It involves the “tendency to react negatively on an emotional, cognitive, and behavioral level to uncertain situations and events” (Buhr and Dugas 2009). Importantly it is a transdiagnostic construct associated with a range of anxiety disorders. Intervention studies with neurotypical individuals provide evidence that a reduction in IU is associated with reduction in anxiety. IU is linked to a range of anxiety disorders including generalised anxiety disorder (GAD) (Buhr and Dugas 2006, 2009, 2012; Dugas et al. 1997, 2005; Freeston et al. 1994), social anxiety (Boelen and Reijntjes 2009; Carleton et al. 2010), panic (Boswell et al. 2013) and anxiety sensitivity more generally (Carleton et al. 2007). Recent headway has been made in investigating its role in typically developing adolescents (Laugesen et al. 2003; Perrin 2014; Wild et al. 2014) and children (Fialko et al. 2012; Kertz and Woodruff-Borden 2013). Cognitive behavioural treatments that emphasise treating the cognitive *process* rather than the cognitive *content* of anxiety, specifically by aiming to increase patients’ tolerance for uncertainty achieve more sustainable change (Wilkinson et al. 2011). Research has confirmed the utility of such protocols in reducing anxiety in adults (Dugas and Ladouceur 2000; Ladouceur et al. 2000; Dugas et al. 2003) and with children and adolescents without ASD (Leger et al. 2003; Payne et al. 2011).

Is IU important in ASD? Over the last 5 years, we have investigated the relevance of IU to ASD. Beyond the evident appropriateness of applying models found to be useful in typically developing populations, the concept itself resonates clinically with some of the core characteristics of ASD (Rodgers et al. 2012). Restricted and repetitive behaviours (RRB), such as insistence on sameness, inflexible adherence to routines and difficulty tolerating change have been linked with anxiety since the earliest descriptions of the disorder (Kanner 1943) and bear a conceptual resemblance to IU, with its associated avoidance of unexpected events and the desire to make life as predictable as possible. Our programme of work provides evidence that IU may have a central role to play in the relationship between ASD and anxiety. Boulter et al. (2014) modelled the relationship between anxiety and IU in an ASD group and a typically developing comparison group. Results confirmed significant relationships between IU and anxiety in children with ASD and were consistent with a causal model suggesting that IU mediates the relationship between ASD and anxiety. Wigham et al. (2015), examined the role of IU in pathways between sensory processing abnormalities, anxiety and RRB in ASD. These relationships were mediated by IU, indicating the important role IU may have in the interaction between anxiety and ASD traits. Chamberlain et al. (2013) report associations between shared neurobehavioural mechanisms in ASD and anxiety, indicating specific avenues for intervention targeting IU. Rodgers et al. (2016) in a study of the development and initial validation of a self and parent report measure of anxiety for youth with ASD (the ASC-ASD) using factor analytic techniques identified four valid anxiety subscales, including an *uncertainty* scale. Hodgson et al. (2016) undertook focus groups with parents of young people with ASD exploring the concept of IU. Parents differentiated IU from dislike of change and fear, discussed examples of IU and its impact in their children and suggested that IU is a recognisable and important construct associated with anxiety distinguishable from but related to features of ASD.

The concept of IU in ASD is now also beginning to be investigated by other groups. Keefer et al. (2015) in a multisite US study demonstrated that in a group of young people with ASD receiving treatment for anxiety, high levels of pre-treatment IU significantly contributed to poorer treatment response. Neil et al. (2016) reported that IU is a relevant construct to sensory sensitivities and anxiety in children with autism. Kerns et al. (2014) in a discussion of the differential diagnosis of anxiety disorders in autism report that fears associated with uncertainty may be an important mechanism in the development and maintenance of anxiety in ASD. Taken together, this evidence indicates that IU is an important mechanism in the development and maintenance of anxiety for young people with ASD and an appropriate target for intervention. However there is currently no treatment available targeting IU in ASD.

A range of cognitive behaviour therapy (CBT) programmes for young people with anxiety and ASD have been evaluated (Chalfant et al. 2007; White et al. 2009; Wood et al. 2009; McConachie et al. 2013), with generally moderate effect sizes. The development of these intervention programmes, driven by increasing awareness of the mental health needs of this population, is in advance of clear understanding of the underlying mechanisms inherent in anxiety in ASD. Given the centrality of IU to anxiety in ASD, we judged it appropriate to develop a parent based group intervention that provides parents of young people with ASD with strategies to reduce IU in their children in everyday situations. Working through parents is appropriate for young people with ASD because it provides parents with strategies that they can utilise with their child across a range of everyday contexts and supports generalisation of these strategies outside of the clinic setting. Parenting a child with ASD is associated with increased parental stress (Hayes and Watson 2013) and this is likely exacerbated if the child is also experiencing anxiety, we therefore also assessed the impact of the intervention on parent wellbeing.

The current study had the following aims:

- To develop a parent based group intervention to specifically address IU in children with ASD.
- To evaluate the feasibility and acceptability of the intervention.

## Method

### Stage 1

Stage 1 of the study involved consultation with parents of young people with ASD and professionals working with young people with ASD in research, clinical or educational settings, about IU. This endeavour took the form of three consultation groups, one for professionals and two for parents. Newcastle University ethics committee provided a favourable ethical opinion for this consultation work. All participants provided informed consent. Parents of children over the age of 8 years old with ASD were invited to attend a focus group through local autism networks, to share their experiences of IU for their child. Nine parents were recruited; three women in the first focus group and six women in the second focus group. Nine professionals working across a range of settings with young people with ASD attended a separate group where they discussed their experiences of IU for the children with ASD they have worked with.

#### Materials

The consultation groups were audio recorded to assist with transcription and analysis. The groups were facilitated by the first and second authors. The parents groups took place at a specialist school in Newcastle, UK and at a community setting in North Tyneside, UK. The professional’s focus group took place at Newcastle University, UK. The primary purpose of the groups was to inform the development of the intervention programme, including (1) checking the acceptability of some of the activities and language that would be used during the intervention, (2) gathering examples from parents about the phenomenology of IU in children with ASD and (3) obtaining further information about strategies that might currently be used to manage IU in children with ASD. A full account of the procedure and findings of the groups can be found in Hodgson et al. (2016).

### Phase 2

Based on findings from Stage 1 and previous work by the research group (Boulter et al. 2014; Wigham et al. 2015; Rodgers et al. 2016) an eight session, manualised, parent group based intervention aimed at reducing IU in young people with ASD was developed ‘Coping with Uncertainty in Everyday Situations—CUES©’.

CUES© aims:

- To develop the young person’s autonomy through the promotion of flexibility and tolerance to everyday uncertainty
- To enable the child to become more able to tolerate uncertainty, rather than attempting to reduce uncertainty
- To identify less helpful strategies that maintain IU and reduce their use by providing an alternative
- To enable parents to work in a zone of proximal development to support their child
- To encourage reflection and evaluation

### Content of the Programme

CUES© is designed to be delivered by community based professionals with knowledge and experience of working with young children with ASD and their families. Two therapists facilitated each group. The first group was delivered by the first author and AH, who is qualified and experienced in Low Intensity CBT. The second group was delivered by the first author and KS, a trainee clinical psychologist.

The groups took place weekly for 8 weeks with each session 2 h in duration. ‘At home’ activities were set each week for parents and children to complete between sessions. The programme began with a focus on the development of understanding of the nature and impact of IU and promoted the use of strategies to flexibly manage IU across a range of settings. The intervention includes psychoeducation to help parents recognise IU, enables parents to identify potential developmental and environmental factors that may trigger IU for their child, and teaches parents to plan and use appropriate strategies aimed at increasing their child’s tolerance of uncertainty. Each parent was provided with a manual in the form of weekly materials and individual support to identify strategies to address a chosen target IU situation. This target situation was the focus for parents to practise the new strategies with their child, thus ensuring that strategies were individually tailored for each child and developmentally appropriate.

The intervention incorporated and synthesised components of existing good practice in relation to anxiety treatment for young people with ASD, including for example the use of comic strips and visual prompts. There were extensive opportunities for mutual learning and support. The group provided the opportunity for parents to develop an understanding of IU and its impact, to try out various strategies to work towards an increasing tolerance of uncertainty for their child and provides opportunities for discussion, mutual support and sharing of ideas, experiences and strategies, importantly building parents’ knowledge and confidence to support their child to develop a more flexible approach to uncertainty.

### Participants

Phase Two of the study involved the delivery of the intervention to fourteen parents across three separate 8 week programmes. The first two courses were delivered to parents recruited via an ASD research database (DASLnE) and were completed between March and June 2014 (n = 3 and 5 parents respectively). The data from these participants has been combined and is referred to hereafter as *Group 1* data. The mean age of the children from Group One whose parents completed the programme (n = 6) was 11 years and 8 months and four were boys. In order to determine whether the programme was acceptable and feasible to families currently receiving services the final group was delivered to five parents recruited via NHS Child and Adolescent Mental Health Services between February and May 2016, hereafter referred to as *Group 2*. The mean age of the children from Group Two whose parents completed the programme (n = 5) was 10 years and 9 months and four were boys. Inclusion criteria were child diagnosis of ASD with no co-occurring intellectual disability, aged between 8 and 15 years, 11 months.

A favourable ethical opinion for phase two was provided by Cornwall and Plymouth NRES Committee South West. All participants provided informed consent.

### Outcome Measures

At the beginning of the programme and at the end of the final session parents completed the following outcome measures:

#### Intolerance of Uncertainty Scale

Parent version (IUS-P; Boulter et al. 2014). The IUS-P is a 12-item questionnaire that assesses IU by asking respondents to rate, on a five point Likert scale, the extent to which statements relating to emotional, cognitive and behavioural responses to IU are like their child.

#### Spence Children’s Anxiety Scale

Parent version (SCAS-P; Nauta et al. 2004)—Group one only. This is a parent-reported Likert scale for children aged 6–18 years. It has 38 items regarding specific anxiety symptoms which parents rate according to frequency from 0 (never) to 3 (always).

#### Anxiety Scale for Children-ASD

Parent version (ASC-ASD, Rodgers et al. 2016). This measure was not available at the time of delivery of the programme to Group 1 and was used with Group 2 only. This is a newly developed anxiety measure for use with children with ASD, with good validity and reliability.

#### Depression Anxiety Stress Scales

Short version (DASS-21; Lovibond and Lovibond 1995). Parents completed the DASS to measure their symptoms of depression, anxiety and tension/stress. Total scores are reported here.

#### Intolerance of Uncertainty Scale

Self report (IUS-12; Carleton et al. 2007) The IUS-12 is a 12-item self-report questionnaire that assesses IU by asking respondents to rate, on a five point Likert scale, the extent to which statements relating to emotional, cognitive and behavioural responses to IU are like them. Parents reported on their own IU.

### Acceptability and Feasibility Outcomes

Parents also completed an evaluation form at cessation of the programme. In addition to the written feedback the six parents from Group One also completed a one to one semi-structured interview 2 weeks after cessation of the programme.

## Results

### Feasibility and Acceptability

Retention to the programme was good across all three groups, with two parents dropping out due to a change in work circumstances preventing them being able to attend the daytime groups (both from Group 1). The baseline scores for these participants were comparable to those who completed the course. Attendance at the sessions was good with 97 % attendance across all three groups.

Parents were also given the opportunity to provide written feedback (Table 1).

**Table 1:** Results from parent completed CUES© evaluation form (n = 12)

Question	Average response (0–4 where 4 indicates most improvement/satisfaction)
How useful was the course?	4
How much has the course increased your understanding of intolerance of uncertainty?	4
How satisfied were you with the course?	4
How much has the course increased your ability to manage your child’s reaction to uncertainty whilst in his/her target situation?	3.7
Have you been able to use the knowledge you’ve acquired through the course when managing your child’s reaction to uncertainty in other situations?	3.8

A summary of some of the comments is provided below:

**In your opinion, what were the best aspects of the course?**

The strategies shared to present positive choices to [child] to help her tolerate uncertainty

Realising uncertainty as well as change affects xxx

Identifying the difference between uncertain situations and change in routine. Trying different strategies in real situations daily. Increasing her tolerance.

**In your opinion, what were the worst aspects of the course?**

None! (×9)

Amount of homework on top of already stressful lives BUT worth it in the end. Progress cannot be made without it. Future parents will need to be made aware of amount of dedication required.

**Has your child’s reaction to uncertainty changed since you started the course? If so, how?**

Yes. He is beginning to independently use relaxation techniques and self calming.

Because I as a parent have had more understanding of uncertainty, my son has felt assured that I have understood him more. I have also been able to explain the reasons for certain behaviours to him and he has now started to manage and recognise his own tool kit of strategies to apply when necessary.

**What is(are) the most useful strategy(ies) you have learnt?**

All have been useful to offer choices

Planning, but not too much due to things can change

Listen to my child. Trust him more in uncertain situations. Review the situation.

Role play, social story, breathing, cue cards

**Would you recommend this course to other parents?**

Yes (11 responses), No (0 responses).

In addition to the written feedback the six parents from Group One also completed a one to one semi-structured interview 2 weeks after cessation of the programme. During the interview parents commented on the impact of attending the CUES programme. Their comments are detailed below:

‘My child is now sleeping better because we now talk about anxiety after dinner rather than before bed, which I learnt on the course.’

My child is now beginning to smile more, which is a big change.

‘We had a meeting with the school last week and although I didn’t use the term ‘intolerance of uncertainty’, I was able to talk to them about his anxiety and explain it better as the ‘fear of the unknown’.

‘It (CUES) worked well for me to attend the sessions, then share the learning with my husband. We have both implemented strategies that I learnt on the course.’

‘My child’s difficulties have not been solved through the course but they have got better through the course, which is great.’

I think it would be helpful to do this with teachers as well so there is a consistent approach.’

### Outcome Measures

Whilst the goal of this study was not to determine the effcicay of the CUES© programme, data in relation to potential outcome measures for future trials was collected and is reported here. The means and standard deviations for all outcomes measures were calculated for each group at baseline and cessation of the programme. Pre and immediate post treatment effect sizes (cohen’s *d*) are also reported (see Table 2).

**Table 2:** Descriptive statistics and effect sizes (cohen’s *d*) for all outcome measures

Study	Gp	n	Mean—pre	SD—pre	Mean—post LOCF	SD—post	ES (based on pooled SD)	Pooled ES
Child								
IUS-P	1	6	48.6	3.9	46.00	5.4	0.56	0.43
	2	5	28.33	10.6	25.00	10.44	0.31	
SCAS	1	6	57.5	18.31	49.5	22.9	0.38	0.43
ASC-ASD	2	5	28.30	10.60	23.30	9.80	0.49	
Parent								
IUS-12	1	6	28.80	7.08	20.80	5.30	1.28	0.83
	2	5	50.20	13.80	45.00	13.00	0.38	
DASS	1	6	26.00	11.30	17.00	10.30	0.83	0.57
	2	5	23.70	18.40	18.80	12.65	0.31	

## Discussion

The aim of this report is to outline the development and preliminary evaluation of the feasibility and acceptability of a parent group based intervention for young people with ASD that focuses on IU. Given the growing evidence base of the centrality of IU to anxiety presentations in individuals with ASD (Chamberlain et al. 2013; Boulter et al. 2014; Wigham et al. 2015; Keefer et al. 2015), coupled with the high prevalence of multiple anxiety disorders concurrently in ASD, targeting important transdiagnostic mechanisms, such as IU, may have significant treatment utility. The study represents the first step in this process, Through consultation with parents of young people with ASD and professionals with clinical, research or educational expertise in autism we developed a manualised, eight session parent group based intervention (CUES©). The programme was then delivered to three groups of parents, two representing community based recruitment and one involving families recruited via clinical services. We collected data relating to acceptability of the programme by recording attendance and completion and through an end of programme evaluation questionnaire. Attendance at and retention to the programme was excellent. Only two parents dropped out. Both of these parents cited changes to work patterns as the reason for their withdrawal and indicated that had they been able to continue attendance they would have done so. All parents who completed the programme indicated that they would recommend the course to other parents and levels of satisfaction and perceived increase in confidence and knowledge in relation to IU were extremely favourable. Parents were also able to provide free text comments in relation to the programme and a sample of these are provided here (the full data set is available on request from the corresponding author) and six participated in a semi-structured interview 2 weeks after the programme addressing the perceived impact of the programme. It can be seen that the parents valued the programme, recognised the role of IU in their child’s lives and found the strategies helpful.

Of course, given the stage of this work in the research cycle the focus here was not on determining the efficacy of the programme. This is a task for future studies. However we were interested in determining the utility of potential outcome measures for use in future trials. To this end parents completed proxy measures of their child’s IU and anxiety and of their own IU and mental health at the beginning and cessation of the programme. The small sample size precludes any inferential statistical analysis of these data, however for information we present the descriptive data here with effect sizes (cohens *d*). Off interest are the gains seen in terms of parent reported child anxiety and parent self-report IU and general mental health. The changes in parent outcomes emphasise the importance of considering parental wellbeing when working with children. Whilst the pooled ES for parent IU-12 and DASS scores indicate medium to large effect sizes across both metrics, there is a striking difference between the ES indicators for the community recruited and clinically referred families, with parents recruited though the community reporting more notable reductions in their IU and DASS scores than the clinically recruited parents. This unanticipated finding is difficult to interpret though it does underscore the importance in future trials of capturing change not just in children with ASD but also in parents who take a central role as agents of change in the programme. Indeed it may be that the intervention could be of value for parents, or as part of a parent management or parent training intervention for ASD. It may help parents to deal with the daily (and long-term) uncertainties that come with having a family member with ASD. This may or may not translate to change in the child (e.g., parents may vary on how well they are able to model IU or to implement teaching strategies to their child); however, it still could have a positive impact on the family overall, which of course then also positively impacts the child. This possibility requires further investigation.

It is of note that although we report a reduction in parent reported child IU for both groups over the course of the programme the effect sizes are modest (d = .56 and .31 respectively with a pooled ES of 0.43). At first glance this may seem disappointing however reflection on the goals of the programme and the timing of the post group outcome measurement may provide a more optimistic interpretation of these findings. Many parents report prior to engagement in the programme that the main strategies used to reduce IU is the avoidance of uncertain situations or attempting to build increased certainty around specific activities (Hodgson et al. 2016). Through engagement in the programme we are supporting parents to expose their child to everyday uncertain situations and/or reduce certainty building strategies and instead utilise strategies to increase tolerance to that uncertainty. It is unrealistic perhaps to expect large treatment gains immediately after completion of the programme, indeed it would not be unanticipated to expect IU to temporarily increase for some young people as parents seek out everyday uncertain situations within which to practise strategies. In this context the small reduction in child IU in the context of exposure to more uncertainty may be a promising indicator of the utility of the programme. In considering these data it is important to note the difference on the baseline IUS-P in Group 1 vs. Group 2. Given the small sample it is difficult to determine the source of this variability. The range of scores on the IUS-P was greater in Group 2 (18–39 vs. 46–56 respectively). At the moment there are no indicative or clinical cut-offs for the IUS-P. It would be important for work to be undertaken in anticipation of future trials to suitable levels of IU for entry into a clinical trial. The lack of longer term follow-up, whilst beyond the scope and goals of the current project is a major limitation of the current study. Of course, what is needed to determine the efficacy of CUES© is a fully powered trial with long-term follow up of both proximal as well as more distal outcomes (e.g. quality of life, family functioning), to determine the clinical impact of the programme. With this in mind it will be important, for future trials, to think very carefully about the nature and timing of outcome assessments and to develop a valid behavioural measure of IU to reduce the reliance on questionnaire measures of IU. It is also important to acknowledge that all of the data collected here were parent report. Given the early stage of the development of the programme our focus was largely on feasibility and acceptability outcomes and no child self-report data were gathered. It will be important to collect data directly from young people themselves in future work. In addition it will be critical in future work to determine if there is a relationship between parent self-report of IU and their rating of their child’s IU low. Of course, it is difficult to note how one would interpret such an association. It may be a consequence of shared method variance, an indicator of the putative heritability of IU or a learned response to uncertainty due to parent (or child) modelling. Indeed through discussion with parents many report being more intolerant of uncertainty over time due to concerns about their child’s reaction to uncertain situations. Furthermore, it is critical in future studies to assess the enactment of the programme by parents outside of the sessions. Tracking these process variables for example, whether parents are positioning their child through the homework tasks to encounter more uncertainty or whether they are providing less certainty in routine situations, will be important information in trying to disentangle the mechanism of change.

In summary the current study sought to take the first steps towards the development of an intervention programme for young people with ASD, which focuses on an transdiagnostic construct underlying anxiety, IU. The preliminary evaluation of the acceptability and feasibility of the novel CUES© programme indicates that the programme is feasible to deliver in both community and clinical settings, acceptable and face valid to parents and effect size estimates of potential outcome measures indicate a fully powered trial is desirable. The current study has provided a valuable opportunity for reflection on next steps and indicated that further work is needed in relation to the nature and timing of outcome measures. It is early days in this work but it would seem that IU is a valid target for treatment programmes aiming to reduce anxiety in young people with ASD.
